# Poly[[tetra­aquadi-μ_4_-fumarato-μ_2_-oxalato-dierbium(III)] tetra­hydrate]

**DOI:** 10.1107/S160053681205026X

**Published:** 2012-12-15

**Authors:** Qing-Feng Yang, Xiao-Zhong Wang, Ping Xue

**Affiliations:** a Key Laboratory of Energy Resources and Chemical Engineering, Ningxia University, Yinchuan 750021, Ningxia, People’s Republic of China; b College of Chemistry and Chemical Engineering, Ningxia University, Yinchuan 750021, Ningxia, People’s Republic of China

## Abstract

The title compound, {[Er_2_(C_4_H_2_O_4_)_2_(C_2_O_4_)(H_2_O)_4_]·4H_2_O}_*n*_, was synthesized by the reaction of erbium nitrate hexa­hydrate with fumaric acid and oxalic acid under hydro­thermal conditions. The Er^3+^ cation (site symmetry ..2) is eight-coordinated by six O atoms from four fumarate anions (site symmetry ..2) and one bidentate oxalate anion (site symmetry 222), and by two water mol­ecules. The complex exhibits a three-dimensional structure consisting of oxalate pillared Er–fumarate layers with channels occupied by coordinating and lattice water mol­ecules. The three-dimensional structure features by O_water_—H⋯O hydrogen bonds involving both the coordinating and lattice water mol­ecules.

## Related literature

For lanthanide–metal complexes containing fumarate ligands, see: Zhang *et al.* (2006 ▶). For lanthanide-containing structures with metal-organic frameworks and two different flexible carboxyl­ate ligands, see: Zhang *et al.* (2008 ▶); Zhu *et al.* (2007 ▶).
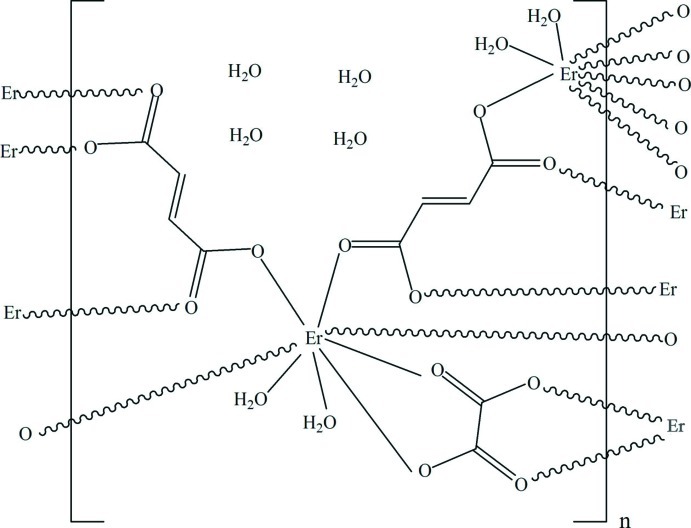


## Experimental

### 

#### Crystal data

[Er_2_(C_4_H_2_O_4_)_2_(C_2_O_4_)(H_2_O)_4_]·4H_2_O
*M* *_r_* = 794.78
Orthorhombic, 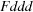

*a* = 9.6016 (19) Å
*b* = 15.701 (3) Å
*c* = 26.722 (5) Å
*V* = 4028.5 (14) Å^3^
*Z* = 8
Mo *K*α radiation
μ = 8.38 mm^−1^
*T* = 293 K
0.19 × 0.16 × 0.13 mm

#### Data collection

Bruker SMART APEX CCD diffractometer
Absorption correction: multi-scan (*SADABS*; Bruker, 2001 ▶) *T*_min_ = 0.305, *T*_max_ = 0.402
9284 measured reflections
1162 independent reflections
1088 reflections with *I* > 2σ(*I*)
*R*_int_ = 0.022

#### Refinement

*R*[*F* ^2^ > 2σ(*F* ^2^)] = 0.018
*wR*(*F* ^2^) = 0.045
*S* = 1.11
1162 reflections
74 parameters
H-atom parameters constrained
Δρ_max_ = 0.51 e Å^−3^
Δρ_min_ = −1.04 e Å^−3^

### 

Data collection: *SMART* (Bruker, 2007 ▶); cell refinement: *SAINT* (Bruker, 2007 ▶); data reduction: *SAINT*; program(s) used to solve structure: *SHELXS97* (Sheldrick, 2008 ▶); program(s) used to refine structure: *SHELXL97* (Sheldrick, 2008 ▶); molecular graphics: *DIAMOND* (Brandenburg, 1999 ▶); software used to prepare material for publication: *SHELXTL* (Sheldrick, 2008 ▶).

## Supplementary Material

Crystal structure: contains datablock(s) I, global. DOI: 10.1107/S160053681205026X/hg5272sup1.cif

Structure factors: contains datablock(s) I. DOI: 10.1107/S160053681205026X/hg5272Isup2.hkl

Additional supplementary materials:  crystallographic information; 3D view; checkCIF report

## Figures and Tables

**Table 1:** Hydrogen-bond geometry (Å, °)

*D*—H⋯*A*	*D*—H	H⋯*A*	*D*⋯*A*	*D*—H⋯*A*
O4—H4*A*⋯O5^i^	0.85	2.35	3.045 (5)	139
O4—H4*B*⋯O3^ii^	0.85	1.91	2.750 (3)	168
O5—H5*A*⋯O4	0.85	1.99	2.836	180
O5—H5*B*⋯O2^iii^	0.85	2.36	2.938 (4)	125

Symmetry codes: (i) 
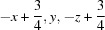
; (ii) 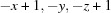; (iii) 
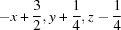
.

**Fig. 1.**
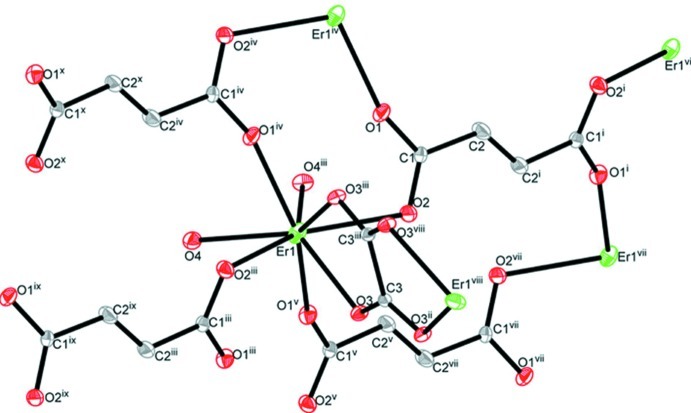
Coordination environment of Er1 in compound I with labelling and displacement ellipsoids drawn at the 30% probability level. Symmetry codes: (i) 2.25 - x, 0.25 - y, z; (ii) 1.25 - x, y, 1.25 - z; (iii) 1.25 - x, 0.25 - y, z; (iv) 1.5 - x, 0.5 - y, 1 - z; (v) -0.25 + x, -0.25 + y, 1 - z; (vi) 1 + x, y, z; (vii) 2 - x, -y, 1 - z; (viii) x, 0.25 - y, 1.25 - z; (ix) -1 + x, y, z; (x) -0.75 + x, 0.25 + y, 1 - z.

**Fig. 2.**
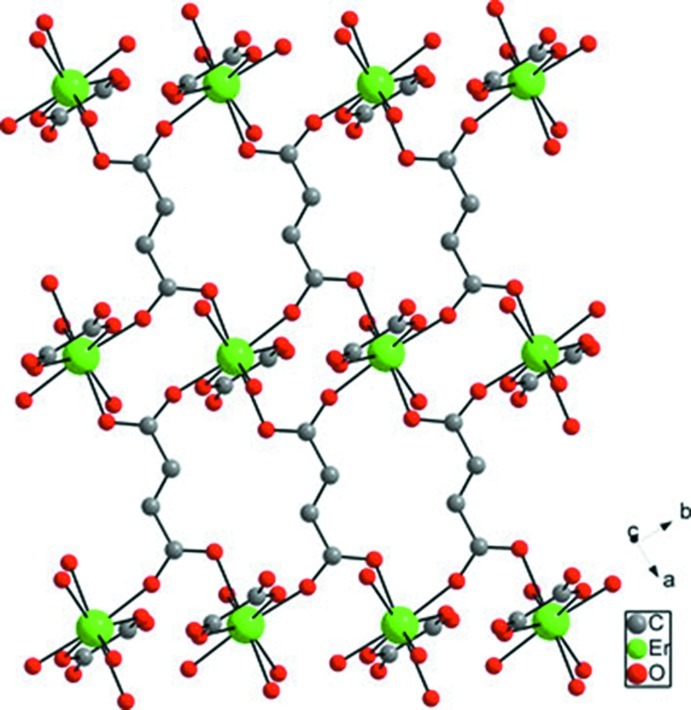
The two-dimensional Er-fum layer extends in the ab plane.

**Fig. 3.**
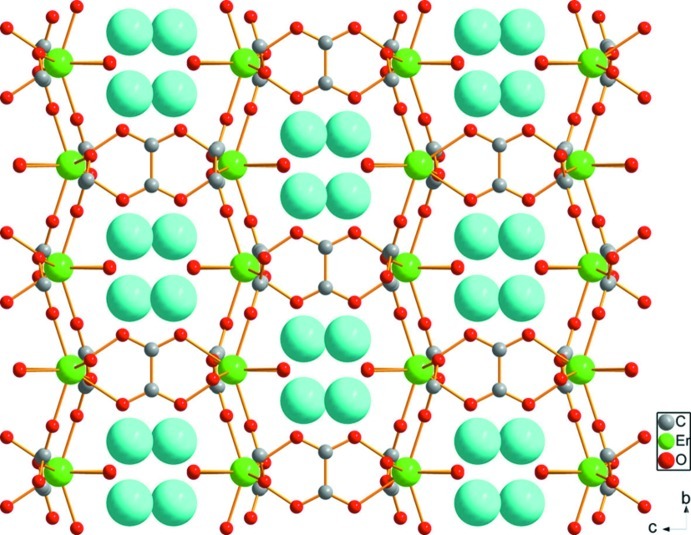
The three-dimensional network with rectangular channels along the [100] direction.

## supplementary crystallographic information

### Comment

In recent years, lanthanide metal-organic compounds have been of great interest due to their fascinating structures and potential applications in magnetism, luminescence, catalysis, gas storage and separation. Multitopic carboxylates have received considerable study due to their availability and potential for allowing for the tailored design of such frameworks. As we know, fumaric acid is a unique ligand with a relatively small, conjugated middle part and versatile coordination modes. A large number of lanthanide metal complexes containing fumarate ligands have been reported, see: Zhang *et al.* (2006). And lanthanide-containing MOFs with two different flexible carboxylate ligands are less developed, see: Zhang *et al.*(2008); Zhu *et al.*(2007). In this paper, we report the synthesis and structure of a new metal-organic compound constructed from fumarate ligands coordinated to Er atoms in the presence of oxalate ligands.

In the title compound I, Er1 is eight-coordinated with four O atoms from four fumarate ligands (O2^iii^, O1^iv^, O2, O1^v^, (iii), 1.25 - *x*, 0.25 - *y*, *z*; (iv) 1.5 - *x*, 0.5 - *y*, 1 - *z*; (v) -0.25 + *x*, -0.25 + *y*, 1 - *z*), two O atoms from one oxalate ligand (O3 and O3^iii^) and two water molecules (O4 and O4^iii^) (Fig. 1). The Er—O bond lengths are between 2.273 (3)–2.428 (3) Å. The Er atoms are linked through bridging carboxyl groups of fumarate ligands to form two-dimensional Er–fum layers in the *ab* plane (Fig. 2). Along the *c* direction, the Er-fum layers are pillared by the oxalic acid resulting in a three-dimensional structure. The framework contains approximately 6.2 Å×11.1 Å rectangular channels along the [100] direction. These channels are occupied by coordinated and free water molecules (Fig. 3). The three-dimensional structure is stabilized by O_water_—H···O hydrogen bonds involving both the coordinated and free water molecules.

### Experimental

A mixture of fumaric acid (0.058 g, 0.50 mmol), oxalic acid (0.063 g, 0.50 mmol) and erbium nitrate hexahydrate (0.230 g, 0.50 mmol) in distilled water (15 ml) was stirred fully in air, and then sealed in 25 ml Teflon-lined stainless steel container, which was heated firstly at 403 K for 2 days and then at 443 K for 1 day. The pink block product, I, was crystallized upon cooling to 273 K.

### Refinement

The H atoms attached to carbon were positioned geometrically and treated as riding on their parent atoms, with C—H 0.93. The hydrogen atoms of the water molecules were located in difference maps and refined by using the '*DFIX*' command with O—H = 0.85 (2)Å with *U*_iso_(H) = 1.5*U*_iso_(O).

### Crystal data

**Table:**

[Er_2_(C_4_H_2_O_4_)_2_(C_2_O_4_)(H_2_O)_4_]·4H_2_O	*D*_x_ = 2.621 Mg m^−^^3^
*M**_r_* = 794.78	Melting point: not measured K
Orthorhombic, *F**d**d**d*	Mo *K*α radiation, λ = 0.71073 Å
Hall symbol: -F 2uv 2vw	Cell parameters from 9875 reflections
*a* = 9.6016 (19) Å	θ = 3.0–27.5°
*b* = 15.701 (3) Å	µ = 8.38 mm^−^^1^
*c* = 26.722 (5) Å	*T* = 293 K
*V* = 4028.5 (14) Å^3^	Block, pink
*Z* = 8	0.19 × 0.16 × 0.13 mm
*F*(000) = 3008	

### Data collection

**Table:**

Bruker SMART APEX CCD diffractometer	1162 independent reflections
Radiation source: fine-focus sealed tube	1088 reflections with *I* > 2σ(*I*)
Graphite monochromator	*R*_int_ = 0.022
Detector resolution: 9.00cm pixels mm^-1^	θ_max_ = 27.5°, θ_min_ = 3.0°
ω scans	*h* = −12→12
Absorption correction: multi-scan (*SADABS*; Bruker, 2001)	*k* = −20→20
*T*_min_ = 0.305, *T*_max_ = 0.402	*l* = −34→31
9284 measured reflections	

### Refinement

**Table:**

Refinement on *F*^2^	Primary atom site location: structure-invariant direct methods
Least-squares matrix: full	Secondary atom site location: difference Fourier map
*R*[*F*^2^ > 2σ(*F*^2^)] = 0.018	Hydrogen site location: inferred from neighbouring sites
*wR*(*F*^2^) = 0.045	H-atom parameters constrained
*S* = 1.11	*w* = 1/[σ^2^(*F*_o_^2^) + (0.0186*P*)^2^ + 60.2587*P*] where *P* = (*F*_o_^2^ + 2*F*_c_^2^)/3
1162 reflections	(Δ/σ)_max_ = 0.003
74 parameters	Δρ_max_ = 0.51 e Å^−^^3^
0 restraints	Δρ_min_ = −1.04 e Å^−^^3^

### Special details

**Table:**

Geometry. All e.s.d.'s (except the e.s.d. in the dihedral angle between two l.s. planes) are estimated using the full covariance matrix. The cell e.s.d.'s are taken into account individually in the estimation of e.s.d.'s in distances, angles and torsion angles; correlations between e.s.d.'s in cell parameters are only used when they are defined by crystal symmetry. An approximate (isotropic) treatment of cell e.s.d.'s is used for estimating e.s.d.'s involving l.s. planes.
Refinement. Refinement of *F*^2^ against ALL reflections. The weighted *R*-factor *wR* and goodness of fit *S* are based on *F*^2^, conventional *R*-factors *R* are based on *F*, with *F* set to zero for negative *F*^2^. The threshold expression of *F*^2^ > σ(*F*^2^) is used only for calculating *R*-factors(gt) *etc*. and is not relevant to the choice of reflections for refinement. *R*-factors based on *F*^2^ are statistically about twice as large as those based on *F*, and *R*- factors based on ALL data will be even larger.

### Fractional atomic coordinates and isotropic or equivalent isotropic displacement parameters (Å^2^)

**Table:**

	*x*	*y*	*z*	*U*_iso_*/*U*_eq_	
C1	0.9358 (4)	0.1660 (2)	0.52974 (13)	0.0167 (6)	
C2	1.0906 (4)	0.1604 (3)	0.53278 (16)	0.0268 (8)	
H2	1.1407	0.2110	0.5348	0.032*	
C3	0.6250	0.0756 (3)	0.6250	0.0150 (8)	
Er1	0.6250	0.1250	0.508677 (7)	0.01528 (8)	
O1	0.8868 (3)	0.23742 (16)	0.51740 (10)	0.0237 (5)	
O2	0.8597 (3)	0.10210 (16)	0.53731 (11)	0.0239 (5)	
O3	0.6164 (3)	0.04009 (15)	0.58332 (9)	0.0190 (5)	
O4	0.4757 (3)	0.12267 (15)	0.43533 (9)	0.0256 (5)	
H4A	0.3985	0.1447	0.4440	0.031*	
H4B	0.4595	0.0706	0.4288	0.031*	
O5	0.5533 (3)	0.20049 (15)	0.34370 (9)	0.0859 (16)	
H5A	0.5298	0.1769	0.3711	0.103*	
H5B	0.5661	0.2534	0.3485	0.129*	

### Atomic displacement parameters (Å^2^)

**Table:**

	*U*^11^	*U*^22^	*U*^33^	*U*^12^	*U*^13^	*U*^23^
C1	0.0141 (15)	0.0186 (16)	0.0173 (16)	0.0032 (13)	−0.0010 (13)	0.0001 (13)
C2	0.0159 (17)	0.0232 (18)	0.041 (2)	−0.0021 (14)	0.0015 (15)	−0.0025 (17)
C3	0.0133 (19)	0.013 (2)	0.019 (2)	0.000	−0.0012 (19)	0.000
Er1	0.01765 (12)	0.01422 (11)	0.01396 (11)	0.00560 (8)	0.000	0.000
O1	0.0239 (13)	0.0200 (12)	0.0270 (13)	0.0067 (10)	0.0003 (11)	0.0045 (10)
O2	0.0168 (12)	0.0205 (12)	0.0344 (14)	0.0009 (10)	0.0001 (11)	0.0027 (11)
O3	0.0269 (13)	0.0139 (11)	0.0161 (11)	−0.0003 (10)	−0.0015 (10)	−0.0010 (9)
O4	0.0296 (13)	0.0215 (12)	0.0256 (13)	−0.0017 (11)	−0.0040 (11)	0.0002 (11)
O5	0.099 (4)	0.076 (3)	0.082 (3)	−0.011 (3)	0.012 (3)	0.037 (3)

### Geometric parameters (Å, º)

**Table:**

C1—O2	1.258 (4)	Er1—O3	2.401 (2)
C1—O1	1.260 (4)	Er1—O2	2.407 (3)
C1—C2	1.491 (5)	Er1—O2^iii^	2.407 (3)
C2—C2^i^	1.294 (8)	Er1—O4	2.428 (2)
C2—H2	0.9300	Er1—O4^iii^	2.428 (2)
C3—O3	1.249 (3)	O1—Er1^iv^	2.273 (3)
C3—O3^ii^	1.249 (3)	O4—H4A	0.8500
C3—C3^iii^	1.550 (9)	O4—H4B	0.8499
Er1—O1^iv^	2.273 (3)	O5—H5A	0.8500
Er1—O1^v^	2.273 (3)	O5—H5B	0.8500
Er1—O3^iii^	2.401 (2)		
			
O2—C1—O1	122.4 (3)	O3—Er1—O2^iii^	77.67 (9)
O2—C1—C2	121.5 (3)	O2—Er1—O2^iii^	142.93 (13)
O1—C1—C2	116.0 (3)	O1^iv^—Er1—O4	74.78 (9)
C2^i^—C2—C1	124.0 (5)	O1^v^—Er1—O4	76.55 (9)
C2^i^—C2—H2	118.0	O3^iii^—Er1—O4	134.38 (9)
C1—C2—H2	118.0	O3—Er1—O4	129.93 (8)
O3—C3—O3^ii^	126.9 (4)	O2—Er1—O4	143.80 (9)
O3—C3—C3^iii^	116.6 (2)	O2^iii^—Er1—O4	72.91 (9)
O3^ii^—C3—C3^iii^	116.6 (2)	O1^iv^—Er1—O4^iii^	76.55 (9)
O1^iv^—Er1—O1^v^	144.28 (13)	O1^v^—Er1—O4^iii^	74.78 (9)
O1^iv^—Er1—O3^iii^	74.25 (9)	O3^iii^—Er1—O4^iii^	129.93 (8)
O1^v^—Er1—O3^iii^	141.35 (9)	O3—Er1—O4^iii^	134.38 (9)
O1^iv^—Er1—O3	141.35 (9)	O2—Er1—O4^iii^	72.91 (9)
O1^v^—Er1—O3	74.25 (9)	O2^iii^—Er1—O4^iii^	143.80 (9)
O3^iii^—Er1—O3	67.62 (11)	O4—Er1—O4^iii^	72.38 (12)
O1^iv^—Er1—O2	106.62 (9)	C1—O1—Er1^iv^	160.8 (3)
O1^v^—Er1—O2	84.78 (9)	C1—O2—Er1	111.9 (2)
O3^iii^—Er1—O2	77.67 (9)	C3—O3—Er1	119.4 (2)
O3—Er1—O2	71.66 (9)	Er1—O4—H4A	106.7
O1^iv^—Er1—O2^iii^	84.78 (9)	Er1—O4—H4B	106.7
O1^v^—Er1—O2^iii^	106.62 (9)	H4A—O4—H4B	106.7
O3^iii^—Er1—O2^iii^	71.66 (9)	H5A—O5—H5B	109.5

Symmetry codes: (i) −*x*+9/4, −*y*+1/4, *z*; (ii) −*x*+5/4, *y*, −*z*+5/4; (iii) −*x*+5/4, −*y*+1/4, *z*; (iv) −*x*+3/2, −*y*+1/2, −*z*+1; (v) *x*−1/4, *y*−1/4, −*z*+1.

### Hydrogen-bond geometry (Å, º)

**Table:**

*D*—H···*A*	*D*—H	H···*A*	*D*···*A*	*D*—H···*A*
O4—H4*A*···O5^vi^	0.85	2.35	3.045 (5)	139
O4—H4*B*···O3^vii^	0.85	1.91	2.750 (3)	168
O5—H5*A*···O4	0.85	1.99	2.836	180
O5—H5*B*···O2^viii^	0.85	2.36	2.938 (4)	125

Symmetry codes: (vi) −*x*+3/4, *y*, −*z*+3/4; (vii) −*x*+1, −*y*, −*z*+1; (viii) −*x*+3/2, *y*+1/4, *z*−1/4.
